# Transient Premature Ovarian Insufficiency Post-COVID-19 Infection

**DOI:** 10.7759/cureus.37379

**Published:** 2023-04-10

**Authors:** Colleen N Gorman, Tori E Abdalla, Yasmina Sultan, Spencer A Grabois, Ellen G Wood

**Affiliations:** 1 Obstetrics and Gynecology, Nova Southeastern University Dr. Kiran C. Patel College of Osteopathic Medicine, Davie, USA; 2 Medicine, Philadelphia College of Osteopathic Medicine, Philadelphia, USA; 3 Biomedical Sciences Program, Philadelphia College of Osteopathic Medicine, Philadelphia, USA; 4 Obstetrics and Gynecology, Mount Sinai Medical Center, Miami, USA; 5 Reproductive Endocrinology and Infertility, IVFMD South Florida Institute for Reproductive Medicine, Cooper City, USA

**Keywords:** corticosteroid, in vitro fertilization (ivf), oophoritis, anti-ovarian antibody, covid-19

## Abstract

Anti-ovarian antibodies (AOAs) have been linked to autoimmune premature ovarian insufficiency (POI). This report details a case in which a patient experienced transient POI after a COVID-19 infection and tested positive for AOA. After treatment with oral contraceptives and subsequent high-dose oral corticosteroids, the patient underwent fertility treatment with in vitro fertilization (IVF). A total of 23 oocytes were retrieved. Two euploid blastocysts and three untested blastocysts were successfully created. This report hypothesizes the connection between autoimmune POI, AOA, and COVID-19. Conflicting data have been reported linking COVID-19 and ovarian injury. However, it is suggested that COVID-19 transiently impacts the menstrual cycle and anti-Mullerian hormone (AMH) levels. Treatment to overcome poor ovarian response due to AOA has not been adequately determined; however, similar autoimmune conditions have been successfully treated with corticosteroids.

## Introduction

Premature ovarian insufficiency (POI) is described as the loss of ovarian function before age 40. The condition is characterized by the absence of menstrual cycles, increased gonadotropins such as follicle-stimulating hormone (FSH) and luteinizing hormone (LH), and decreased estradiol (E2) levels [1]. A multitude of causes have been identified in association with POI, such as genetic, chromosomal, iatrogenic, autoimmune, and infectious [1]. The exact mechanisms behind the autoimmune causes of POI are still being investigated; however, evidence of the association between POI, the presence of anti-ovarian antibodies (AOA), autoimmune oophoritis, and preexisting autoimmune diseases have been reported [1,2].

Autoimmune conditions, such as autoimmune thyroid disease, type one diabetes mellitus, and systemic lupus erythematosus, have been linked to decreases in anti-Mullerian hormone (AMH), a hormone produced by the pre-antral and small antral follicles within the ovary [2]. AMH is considered a predictor of ovarian reserve and, if decreased, is associated with poor response to ovarian stimulation [3]. AOA, along with other cellular immunity dysfunction, might have a connection to the autoimmune process behind POI [4]. AOAs have been associated with decreased pregnancy rates in women undergoing infertility treatments [5].

In the face of normal FSH and LH levels, alternate causes must be investigated when an unanticipated response to ovarian stimulation occurs. An infection could be a potential etiology for a sudden abnormal ovarian response. Due to the most recent COVID-19 pandemic and lack of knowledge on the effect of the novel virus on reproduction, a correlation between a recent COVID-19 infection and an abnormal ovarian response needs to be considered. There is currently no literature investigating the association between COVID-19 and AOA; however, anecdotal evidence reports POI in a female patient after testing positive for COVID-19 [6]. The current literature remains unclear about the association between ovarian injury and COVID-19. Some studies have found no statistical difference in AMH levels and, thus, ovarian reserve before and after recovering from COVID-19 [7-9]. It has been hypothesized that the COVID-19 pandemic, treatments, and vaccines may impact the menstrual cycle by affecting the hypothalamic-pituitary-ovarian-endometrial axis [10]. One study reported decreased mature oocytes and the number of oocytes retrieved in patients with higher COVID-19 IgG levels compared to those without antibodies to the virus [11].

Another study suggested a potential mechanism of ovarian injury through the angiotensin-converting enzyme 2 (ACE2) receptor entering the granulosa cells through transmembrane protease serine 2 enzyme (TMPRSS2), leading to a pathological process that decreased AMH levels and increased testosterone and prolactin levels [12]. Finally, a 2022 literature review reported slight changes in AMH levels and significant alterations in menstruation after infection with COVID-19; however, these changes in menstruation appeared reversible [13].

This case report adds to the literature on POI by detailing a case in which AOAs were present after infection with COVID-19 in a patient who was trying to conceive. We hypothesize that COVID-19 possibly caused a transient autoimmune oophoritis demonstrated by positive AOA and hormone levels consistent with POI postinfection with the novel virus. This patient was able to be stimulated for in vitro fertilization (IVF) after suppression with corticosteroids. Ovarian response was achieved, and euploid blastocysts were created.

## Case presentation

Patient history

In December 2021, a 27-year-old female G0P0 presented to the clinic with primary infertility since stopping birth control pills in March 2021. Her menstrual cycles were irregular before starting birth control pills but were regular six months after discontinuing the medication. She denies dysmenorrhea or dyspareunia. Her past medical history includes intermittent asthma, anxiety, and a hiatal hernia. She reports multiple environmental allergies and a significant amount of food sensitivities. Her medications include prenatal vitamins, Vitex, Symbicort, Montelukast, Omeprazole, Sertraline, and weekly allergy shots. Upon presentation, she had received the first dose of the Pfizer COVID-19 vaccine in August of 2021 and had not been infected with COVID-19 prior. She did not receive the second Pfizer COVID-19 vaccine. Her past surgical history includes breast augmentation. Her family history includes two paternal aunts requiring fertility treatment, one with IVF and another with ovulation induction with clomiphene citrate. She is a social alcohol drinker and denies tobacco and recreational drug use. She works as a second-grade teacher.

Partner history

A 27-year-old male without proven fertility reports no past medical history. His only medication is male fertility vitamins. He received the Pfizer COVID-19 vaccine in August of 2021 and had never been infected with COVID-19. He has no allergies and a negative surgical history. He denies alcohol and tobacco use and stopped using marijuana three months before the fertility evaluation. He works as a boat mechanic.

Initial fertility evaluation

The patient's initial AMH in December 2021 was 6.59 ng/mL. Her cycle day three FSH was 11.2 mIU/mL, and E2 was 77 pg/mL. Reference values for blood hormone levels include an average AMH of 3.82 ng/mL for a 27-year-old female, and a cycle day three FSH and E2 of 3-20 mIU/mL and 25-75 pg/mL, respectively. Hysterosalpingogram revealed a normal uterus and patent fallopian tubes. Semen analysis revealed a sperm count of 24 million, 56% motility, and 1.5% normal morphology. Reference values for semen analysis include sperm count of 39 million per ejaculate, percent motility of 40%, and 4% normal morphology. The couple was diagnosed with mild male factor infertility and possible ovulatory dysfunction.

Initial fertility treatment

The patient began treatment in March 2022 with 5 mg of letrozole for five days, from cycle day three through day seven, with ultrasound monitoring. A mature follicle developed by cycle day 14, and the patient was triggered with human chorionic gonadotropin (hCG) for a single intrauterine insemination (IUI) cycle. She was inseminated with 10.2 million total motile sperm. Her pregnancy test was negative, and her mid-luteal progesterone level was 11 ng/mL. The patient then contracted COVID-19 after her one unsuccessful letrozole/IUI cycle in June 2022. The patient did not require hospitalization during her illness. She did not receive monoclonal antibody therapy or antiviral treatment while infected. Her partner was not infected with COVID-19. 

Fertility treatment post-COVID-19 infection

Once recovered from COVID-19 in July 2022, the patient began another letrozole cycle with 7.5 mg of letrozole from cycle day three to day seven, yielding no ovarian response with ultrasound monitoring by cycle day 16. Hormone levels were repeated, revealing an AMH of 0.71 ng/mL and an FSH and E2 of 21.6 mIU/mL and 37 pg/mL, respectively. The patient was then tested for AOA and anti-adrenal antibodies in September 2022. The AOA titer came back positive at 1:1,280, and the anti-adrenal antibodies titer was also positive at 1:40. The patient remained amenorrheic. Repeat labs in October 2022 showed an AMH level of 4.66 ng/mL and an FSH and E2 of 9.8 mIU/mL and <20 pg/mL, respectively. The patient was then started on continuous birth control pills for three months.

The couple then decided to move to IVF treatment. The patient stopped taking her birth control pills for five days and was started on gonadotropins. Her E2 level was <20 pg/mL at the start of stimulation. She was then given 225 international units (IU) of FSH and 75 IU of human menopausal gonadotropin (hMG) for seven days, and her E2 remained <20 pg/mL. The medication was then changed to 225 IU of hMG and 75 IU of FSH for three more days, with no ovarian response and an E2 < 20 pg/mL. The injectable drug stimulation was stopped, and the patient was then started on Prednisone 30 mg twice daily.

The patient was started on a delayed stimulation protocol checking E2 levels daily. After seven days of prednisone, her E2 rose to 60 pg/mL, and gonadotropin stimulation was restarted at 150 IU of FSH and 150 IU of hMG for three days. The E2 rose to 136 pg/mL. The FSH was then increased to 225 IU with 150 IU of hMG. 

After 15 days of gonadotropin stimulation, the patient reached a peak E2 of 593 pg/mL and was triggered with an injection of human hCG and a GnRH agonist for a 35-hour transvaginal oocyte retrieval. The prednisone was discontinued the day before the transvaginal oocyte retrieval was performed. Four follicles greater than 18 mm, one 17 mm follicle, three 16 mm follicles, four 15 mm follicles, one 14 mm follicle, and three 13 mm follicles were identified on the day of the trigger injection. The post-trigger E2 was 659 pg/mL, and the post-trigger beta-hCG level was 153 mIU/mL. The luteinizing hormone was 82.8 mIU/ mL, and progesterone was 11.7 ng/mL. She had 23 oocytes retrieved: 11 metaphase two (M2) oocytes, five metaphase one (M1) oocytes, five germinal vesicles, and two degenerated oocytes. Nine oocytes were fertilized normally, yielding two euploid blastocysts and three untested blastocysts that were all cryopreserved. The patient is planning to undergo a frozen embryo transfer.

## Discussion

The relationship between COVID-19 and female fertility has not been clearly defined. While it is known that the receptor for COVID-19, ACE2, is expressed in the uterus and ovaries, reports on fertility outcomes postinfection are limited [11]. Autoimmunity after COVID-19 infection, including the development of AOA, has been reported and could be a primary cause of infertility [13]. While AOAs have been documented in patients before the novel virus, a significant rise in antibody concentration after infection should be a consideration in patients presenting with an inability to conceive.

Patients with AOA undergoing IVF have been primarily treated with corticosteroids. The dose, onset, and duration of corticosteroid use during a cycle varies widely between provider practices. Serum conversion from AOA positive to negative before IVF versus initiation of corticosteroids at the start of stimulation and continued into the pregnancy remains controversial. Using 0.5 mg/kg prednisolone starting the first day of stimulation and continuing until the end of the first trimester has been shown to increase pregnancy, implantation, and live birth rates in AOA-positive patients with two prior failed IVF attempts [14]. Clinicians have also used 0.5 mg dexamethasone twice daily to convert AOA-positive patients to negative before IVF treatment, with clinical pregnancy rates similar to those of AOA-negative patients [15]. Ten milligrams per day of prednisone one month before ovulation induction in AOA-positive patients has also improved embryo grading and pregnancy rates [16].

The effect of autoimmunity on IVF outcomes has been well reported. Thyroid, antiphospholipid, antinuclear, antigonadotropin, anti-endometrial, and anti-laminin-1 antibodies have all been shown to have a negative impact on the success of IVF treatment [17]. The choice of immunotherapy in infertile patients with autoimmune conditions is essential. The mainstay of treatment has included corticosteroids [18], whereas other immunosuppressive agents, such as cyclophosphamide, can cause premature ovarian failure, rendering IVF less effective [19]. 

This report highlights the successful use of corticosteroids in an AOA-positive patient after developing post-acute COVID-19 syndrome. After failing the initial stimulation protocol for IVF, the patient was placed on twice daily prednisone, allowing an initial E2 rise and follicular development to occur. Based on the E2 rise, it was anticipated that only three oocytes were to be retrieved. However, a surprising number of follicles were seen on ultrasound, and multiple oocytes were subsequently retrieved. There is no reported evidence to support the relationship between AOA and POI; however, researchers have hypothesized that AOA antibodies surrounding granulosa cells can act as FSH receptor antagonists and decrease the production of E2 [20]. We propose a possible AOA antibody effect on the granulosa cells, blunting E2 secretion yet not altering oocyte development might exist. Ultimately, this patient had 11 mature oocytes retrieved that created two euploid blastocysts and three untested blastocysts that were cryopreserved.

Patients undergoing IVF treatment receive extensive testing before stimulation, and screening for AOA as a cause of infertility is justifiable in patients with self-reported COVID-19 infection. Doing so can avoid unnecessary workup, save on patient financial investment, and improve IVF effectiveness. Time to clinical pregnancy can be decreased in patients with appropriately identified causes of infertility with proper treatment. It is reasonable to counsel and evaluate AOA in an era of ongoing COVID-19 infection and with the wide use and safety of corticosteroids for its treatment.

## Conclusions

This is a case of transient autoimmune oophoritis resulting in POI after infection with COVID-19, which was successfully treated with corticosteroids. Evaluation of infertility patients with self-report of recent infections should include screening for AOA as a cause of infertility. Early detection of AOA in patients with no other identifiable causes of infertility can decrease the time to clinical pregnancy with cost-effective medication. Management of patients with a positive AOA screening test includes treatment with corticosteroids before and during ovarian stimulation. Evaluating patient titers during stimulation, before and after embryo transfer, and throughout the resulting pregnancy for medication management can be considered.

## References

[1] Stuenkel CA, Gompel A (2023). Primary ovarian insufficiency. N Engl J Med.

[2] Chen J, Wu S, Wang M, Zhang H, Cui M (2022). A review of autoimmunity and immune profiles in patients with primary ovarian insufficiency. Medicine (Baltimore).

[3] Cavalcante MB, Sampaio OG, Câmara FE (2023). Ovarian aging in humans: potential strategies for extending reproductive lifespan. Geroscience.

[4] Košir Pogačnik R, Meden Vrtovec H, Vizjak A, Uršula Levičnik A, Slabe N, Ihan A (2013). Possible role of autoimmunity in patients with premature ovarian insufficiency. Int J Fertil Steril.

[5] Edassery SL, Shatavi SV, Kunkel JP (2010). Autoantigens in ovarian autoimmunity associated with unexplained infertility and premature ovarian failure. Fertil Steril.

[6] Wilkins J, Al-Inizi S (2021). Premature ovarian insufficiency secondary to COVID-19 infection: an original case report. Int J Gynaecol Obstet.

[7] Madendag IC, Madendag Y, Ozdemir AT (2022). COVID-19 disease does not cause ovarian injury in women of reproductive age: an observational before-and-after COVID-19 study. Reprod Biomed Online.

[8] Kolanska K, Hours A, Jonquière L (2021). Mild COVID-19 infection does not alter the ovarian reserve in women treated with ART. Reprod Biomed Online.

[9] Horowitz E, Mizrachi Y, Ganer Herman H (2022). The effect of SARS-CoV-2 mRNA vaccination on AMH concentrations in infertile women. Reprod Biomed Online.

[10] Sharp GC, Fraser A, Sawyer G (2022). The COVID-19 pandemic and the menstrual cycle: research gaps and opportunities. Int J Epidemiol.

[11] Herrero Y, Pascuali N, Velázquez C (2022). SARS-CoV-2 infection negatively affects ovarian function in ART patients. Biochim Biophys Acta Mol Basis Dis.

[12] Ding T, Wang T, Zhang J (2021). Analysis of ovarian injury associated with COVID-19 disease in reproductive-aged women in Wuhan, China: an observational study. Front Med (Lausanne).

[13] Carp-Veliscu A, Mehedintu C, Frincu F (2022). The effects of SARS-CoV-2 infection on female fertility: a review of the literature. Int J Environ Res Public Health.

[14] Forges T, Monnier-Barbarino P, Guillet-May F, Faure GC, Béné MC (2006). Corticosteroids in patients with antiovarian antibodies undergoing in vitro fertilization: a prospective pilot study. Eur J Clin Pharmacol.

[15] Pires ES, Parikh FR, Mande PV, Uttamchandani SA, Savkar S, Khole VV (2011). Can anti-ovarian antibody testing be useful in an IVF-ET clinic?. J Assist Reprod Genet.

[16] Geva E, Fait G, Lerner-Geva L (1999). The possible role of antiovary antibodies in repeated in vitro fertilization failures. Am J Reprod Immunol.

[17] Simopoulou M, Sfakianoudis K, Maziotis E (2019). The impact of autoantibodies on IVF treatment and outcome: a systematic review. Int J Mol Sci.

[18] Gokce S, Herkiloglu D, Ozdemir S, Ozvural S, Karabacak O (2021). The effect of prednisolone treatment on pregnancy rates in in vitro fertilization patients with positive thyroid autoantibodies. Sisli Etfal Hastan Tip Bul.

[19] Tsaliki M, Koelsch KA, Chambers A, Talsania M, Scofield RH, Chakravarty EF (2021). Ovarian antibodies among SLE women with premature menopause after cyclophosphamide. Int J Rheum Dis.

[20] Pires ES (2010). Multiplicity of molecular and cellular targets in human ovarian autoimmunity: an update. J Assist Reprod Genet.

